# Bridging the Gap: Enhancing Prostate Cancer Survivorship and Advocacy Among Ethnically Diverse Black Men Through Community Town Halls

**DOI:** 10.1007/s13187-025-02587-1

**Published:** 2025-02-24

**Authors:** Motolani E. Ogunsanya, Ernest Kaninjing, Sara M. DeForge, Daniel J. Morton, Perry Cole, Patrick Beckford, Donald Reese, Thomas Mitchell, Everett Montgomery, Roland Odeleye, Erick Y. Miró-Rivera, Kaitlin Roberts, Jordan M. Neil, Adam C. Alexander, Rachel K. Funk-Lawler, Desiree Azizoddin, Mack Roach, Samuel L. Washington, Kathleen Dwyer, Mary Ellen Young, Folakemi T. Odedina, Darla E. Kendzor

**Affiliations:** 1 Department of Family and Preventive Medicine, University of Oklahoma Health Sciences, Oklahoma City, OK USA; 2 TSET Health Promotion Research Center, University of Oklahoma Health Sciences, Oklahoma City, OK USA; 3 OU Health Stephenson Cancer Center, University of Oklahoma Health Sciences, Oklahoma City, OK USA; 4 The Multidisciplinary Health Outcomes Research and Economics (MORE) Lab Community Advisory Board, University of Oklahoma Health Sciences, Oklahoma City, OK USA; 5 School of Health and Human Performance, Georgia College & State University, Milledgeville, GA USA; 6 Department of Pediatrics, University of Oklahoma Health Sciences, Oklahoma City, OK USA; 7 School of Pharmacy, Medical Sciences Campus, University of Puerto Rico, San Juan, Puerto Rico, USA; 8 Wingate University School of Pharmacy, Wingate, NC USA; 9 Department of Psychiatry and Behavioral Sciences, University of Oklahoma Health Sciences, Oklahoma City, OK USA; 10 Department of Radiation Oncology, University of California, San Francisco, CA USA; 11 Division of Urology, Divison of Epidemiology & Biostatistics, University of California, San Francisco, CA USA; 12 Fran and Earl Ziegler College of Nursing, University of Oklahoma Health Sciences, Oklahoma City, OK USA; 13 Mayo Foundation for Medical Education and Research, Jacksonville, FL USA; 14 Mayo Clinic Comprehensive Cancer Center, Mayo Clinic, Jacksonville, FL USA; 15 Department of Hematology and Oncology, Mayo Clinic, Jacksonville, FL USA

**Keywords:** Prostate cancer, Town halls, Cancer survivorship, Quality of life, Patient education

## Abstract

We present findings from town hall meetings held with ethnically diverse Black prostate cancer (CaP) survivors in 2022 and 2023. Four town halls (total participants, *N* = 265) focused on diagnosis, treatment, psychosocial support, and overall survivorship. Moderated by a long-term CaP survivor, each session featured a panel that included medical providers (urologic oncologists and clinical psychologists), as well as CaP survivors of American, Caribbean, and African origin and their spouses. Surveys captured demographic data, clinical information, CaP-specific details (for survivors only), and perceptions of the town halls. The meetings highlighted three interrelated themesinfluencing decision-making: access to comprehensive information, the strength of support networks, and the availability of culturally competent care. These town halls revealed important educational and support opportunities for ethnically diverse Black men with CaP, promoting informed decision-making throughout the cancer journey and improving quality of life while reducing decisional regret during survivorship.

**Supplementary Information:**

The online version contains supplementary material available at 10.1007/s13187-025-02587-1.

## Introduction

Prostate cancer (CaP) is the second most common cancer in the USA, with 313,780 new cases and 35,770 deaths predicted in 2025 [1]. Black men (BM) experience a disproportionate burden from CaP, with a younger average age at diagnosis, more frequent presentation with aggressive disease, and higher mortality than non-Hispanic White men (NHWM) [2, 3]. Despite these disparities, the overall 5-year survival for BM with CaP is approximately 97%, leading to a growing population of survivors deal facing numerous physical, emotional, and financial sequelae of the disease [4, 5]. Addressing these quality of life (QoL) challenges through survivorship care and patient advocacy is critical. In recent decades, immigration, principally from Africa and the Caribbean, has dramatically changed Black demographics in America [6]. Thus, to effectively address CaP disparities, it is essential to include a wide range of BM in research, including native-born BM, African-born BM, and Caribbean-born BM, to develop tailored survivorship strategies that account for ethnicity and cultural beliefs which can shape health behaviors and impact QoL [7–10].

Community-based interventions that foster support, awareness, and education are promising strategies for addressing QoL challenges, enhancing CaP management, and reducing healthcare disparities among BM. Among community-based approaches, town halls are valuable forums for open discussion of health care issues, facilitating knowledge sharing and empowering participants with accurate, culturally relevant information [11–13]. Such gatherings can foster trust, bridge communication gaps between patients and providers, and create a comprehensive support network by incorporating healthcare professionals, researchers, community leaders, and spouses alongside CaP survivors [11]. Town halls also offer a safe space for sharing experiences, emotions, and coping strategies, which can profoundly impact psychological well-being and treatment adherence, alleviate social isolation, and empower participants to actively engage in their healthcare [11–13]. We hypothesized that town halls could bridge gaps in CaP survivorship management, and accordingly, this manuscript reports findings from town halls conducted with ethnically diverse Black CaP survivors in 2022 and 2023, focusing on diagnosis, treatment, psychosocial support, and overall survivorship.

## Research Design

### Town Halls

Guided by an agenda (see sample in Table 1; Online Resource *in Supplementary Info*), four virtual town halls, each lasting 1.5 h, were held via Zoom webinars in English, concluding with a Q&A session. All sessions were recorded, transcribed verbatim, and accompanied by detailed notes from research staff. Two town halls included in-person watch parties at churches and barbershops in Milledgeville, GA, and Dallas, TX. Panelists, including survivors, spouses, and healthcare providers (Table 1), discussed predetermined topics moderated by a CaP survivor using a facilitator guide informed by our prior grounded theory evaluation [14]. Topics were initially developed internally and subsequently reviewed by our Community Advisory Board (CAB), which comprises seven Black CaP survivors and a Black primary care provider. The CAB provided input and approved the final topic list. The discussion topics included (1) community concerns about CaP health; (2) survivorship testimonies/experiences; (3) attitudes toward CaP resources, early detection, and treatment; (4) perspectives on health conversations; and (5) mental health in survivorship.All promotional materials were reviewed by the CAB before dissemination to ensure cultural sensitivity and community relevance.

**Table 1:** Town hall meeting information

Town hall topic	Date	Audience	Facilitator	Panelists	Attendees	Surveys completed
1. Stories from survivors and those who love and care for them	April 2022	General public, mixed ages	CBBM, CaP survivor > 20 years	NBBM, radiation oncologist, medical oncologist, and urologist NBBM CaP survivor ABBM CaP survivor CBBM CaP survivor BF, spouse of CaP survivor	53	26
2. Treatment options, mental health considerations, and supportive care for survivors and caregivers	September 2022	General public, mixed ages	CBBM, CaP survivor > 20 years	NBBM, radiation oncologist, medical oncologist, and urologist NBBM CaP survivor ABBM CaP survivor CBBM CaP survivor BF, spouse of CaP survivor (2) Psychologist	81	35
3. Diagnosis journey: overcoming hesitancy, misconceptions, and cultural barriers	June 2023	General public, mixed ages	CBBM, CaP survivor > 20 years	NBBM, radiation oncologist WF, clinical psychologist NBBM CaP survivor NBBM CaP survivor CBBM CaP survivor	72	31
4. Elevating comfortability around health conversations and early diagnosis	November 2023	General public, mixed ages	CBBM, CaP survivor > 20 years	NBBM, urologic oncologist WF, pain psychologist, behavioral scientist NBBM CaP survivor (2) CBBM CaP survivor ABBM CaP survivor	59	25

*CaP* prostate cancer, *NBBM* native-born Black male, *CBBM* Caribbean-born Black male, *ABBM* African-born Black male, *BF* Black female, *WF* White female.

### Recruitment

Participants were recruited via flyers, referrals, and snowball sampling. Social media advertisements were widely employed, and QR-code-embedded flyers (see Figure 2; Online Resource *in* *Supplementary Inf*o) were strategically posted in clinics and predominantly Black businesses, organizations, and community settings. This approach leveraged existing research and community networks, including clinicians, CaP survivors, and their families, as well as community groups. We encouraged community members and physicians to invite eligible participants to attend town halls. Collaboration with the CAB was vital in refining and implementing our recruitment strategy, which aimed to ensure broad outreach and engagement within Black communities [15]. This approach was approved by the University of Oklahoma Health Sciences IRB (11,441) with waived written informed consent.

### Instruments

A post-town hall survey (see Table 2*in Supplementary Info*) collected participant demographics: age, gender, ethnicity, marital status, family history of CaP, connection to CaP, and, for participants with CaP, age at diagnosis, current treatment status, stage of diagnosis, and treatment types. Perceptions of the town hall, including organization and sources of information, were also assessed. Open-ended questions allowed participants to share likes, dislikes, perceived community benefits, reasons for attending the town hall, and suggestions for future topics. Upon survey completion, participants received gift cards; CaP survivors received $25, and others received $10. Panelists (survivors only) and the facilitator received $100 and $300 remuneration, respectively.

### Data Analysis

Descriptive statistics from surveys were obtained using SPSS v27. Inductive thematic analysis was used to analyze the qualitative data via Atlas.ti v20. Team members (MO, EMR, KR, SD) independently reviewed Zoom recordings, observation notes, and transcripts—each member independently coded questions and discussions for semantic meaning, grouping codes into categories and themes. Team members compared and assimilated their codes, categories, and themes to ensure rigor and reliability, engaging in extensive discussions to resolve discrepancies and reach a consensus. Several consensus coding meetings occurred over 4 months. All data were co-coded by team members to improve inter-coder reliability. Data triangulation enhanced validity by integrating findings from multiple sources. Extracts corresponding to each theme were compiled into a matrix to identify recurring themes across all four town halls.

## Results

### Surveys

Of 265 attendees, 44% (*n* = 117) completed follow-up surveys (Table 2). Most participants were male (76.1%) and married (68.4%), and they self-identified as Black: African-American (33.3%), Black: African (29.9%), and Black: Caribbean (12%). Over 40% (*n* = 51) of survey respondents were CaP survivors, with an average age at diagnosis of 59 years, most commonly reporting radical prostatectomy as their treatment (20.5%). Overall, perceptions of the town halls were positive, with 73.4% rating them as excellent or very good and 37% describing them as extremely organized.

**Table 2:** Clinical and sociodemographic characteristics of respondents (*n* = 117^#^)

**Characteristics**	
**Age, years (range, mean ± SD)**	23–86 (48 ± 14)
**Family history of CaP (%)**	
Yes	49 (41.9)
No	46 (39.3)
Unknown/unsure	22 (18.8)
**Sex (%)**	
Male	89 (76.1)
Female	26 (22.2)
**Ethnicity (%)**	
Black: African-American	39 (33.3)
Black: African-born	35 (29.9)
Black: Caribbean-born	14 (12.0)
White or Caucasian	10 (8.5)
Asian or Pacific Islander	6 (5.1)
Hispanic or Latina	4 (3.4)
Native American or Alaskan Native	2 (1.7)
**Marital status (%)**	
Married	80 (68.4)
Single	19 (16.2)
Divorced/widowed/separated	16 (13.7)
**Income (%)**	
< $50 k	39 (33.3)
$50,001–$75,000	33 (28.2)
> $75 k	43 (36.8)
**Education (%)**	
High school graduate or less/GED	2 (1.7)
Some college or technical school	17 (14.5)
College graduate	43 (36.8)
Postgraduate	51 (43.6)
**Relationship with CaP (%)**	
Survivor and advocate	32 (27.3)
Other relative of a survivor (e.g., child, sibling)	21 (17.9)
Survivor	19 (16.2)
Clinician or other healthcare provider	18 (15.4)
Spouse of a survivor	6 (5.1)
Other (scientist, lost a loved one to CaP, concerned community member)	17 (14.5)
*Age at diagnosis, years (range, mean ± SD)	45–78 (59 ± 7)
*Currently undergoing CaP treatment (%)	
Yes	16 (31.4)
No	29 (56.9)
*Stage of diagnosis (%)	
Stage I	19 (37.3)
Stage II	13 (25.4)
Stage III	3 (5.9)
Stage IV	2 (3.9)
Unsure	14 (27.4)
*Treatment type (%)	
Radical prostatectomy	24 (20.5)
Radiotherapy	19 (16.2)
Hormonal	8 (6.8)
Other (e.g., active surveillance)	8 (6.8)
**Overall perception of town hall (%)**	
Excellent	52 (44.4)
Very good	35 (29.9)
Good	9 (7.7)
Fair	2 (1.7)
**Organization of town hall (%)**	
Extremely organized	43 (36.8)
Very organized	39 (33.3)
Somewhat organized	16 (13.7)
**Source of town hall info (%)**	
Word of mouth (including clinicians, CAB members, colleagues)	88 (75.2)
Social media	16 (13.7)
Print media (newspaper ad)	4 (3.4)

^#^Not all respondents answered all questions; thus, totals for all categories do not equal 117. All percentages are calculated based on 117 respondents

*For participants with CaP diagnosis only

Open-ended responses provided strong positive feedback and constructive suggestions for improvements. Participants appreciated the informative sessions, diverse perspectives, and speaker quality. Participants commended the transparency and honesty of panelists, the sense of community fostered during the event, and the efforts to raise awareness and support those affected by the disease. Suggestions for improvement included leaving more time for general discussion, engaging a greater representation of long-term CaP survivors, and dedicating more discussion time to erectile dysfunction and mental health.

### Town Hall Themes

In addition to survey findings, town halls highlighted three significant resources accessed during the CaP experience: information, support networks, and culturally competent care. These resources interacted with the final theme—decision-making—by influencing the timing, content, and emotional aspects of care decisions (Table 3).

**Table 3:** Town hall themes and representative quotes

Theme(s)	Subtheme(s)	Quote
Information	Individual level	*“…at that point, I had to make some quick, fast, hard decisions, knowing nothing about prostate cancer treatment options… know what treatment options are out there before you’re faced with having to make the option of treatment. We should know more about prostate cancer before it affects you instead of having to make the decisions under the gun.”* NBBM, 65 yr old, CaP survivor (TH1) *“My treatment option is active surveillance. And it's not blindly making that decision. I made that decision in consultation and with a lot of information from various sources.”* ABBM, 65 yr old, CaP survivor (TH4)
	Community level	“*…I have two siblings that were… lived in the UK. I reached out to them to let them know, you know, what my diagnosis was. And my sister, who was a nurse, got back to me and told me, well, have your doctor send the report, and I am going to take it to my doctor that I work with at the hospital. Because he is one of the leading doctors in the UK in prostate cancer, I sent her the report, and then she got back to me and said, oh, the doctor advised that if this were her brother, she’d tell him to have a radical prostatectomy. I was leaning towards that because I had books, and I was reading.”* CBBM, 75 yr old, CaP survivor (TH1) *“…once I went public, more and more wives and members… and brothers themselves began to call me, to ask me to talk to either them or their children.”* NBBM, 84 yr old, CaP survivor (TH3)
	Second opinion	*“One of the things that you have to do when you get diagnosed with prostate cancer, depending upon where you’re being managed, is sometimes you have to get a second opinion on the pathology.”* NBBM, urologist (TH1) “*I tell people, I like cutting. I like doing surgery, but it’s got to help you, and it has to be the thing you want. So, if there are multiple treatments, you should hear from each person doing the treatment. Listening to a surgeon about radiation is not going to be the same as listening to a radiation doctor talk about radiation. So, you won’t get the informed decision you need to make a critical decision. So, second opinions, talking to specialists, is standard*.” NBBM, urologist (TH1)
Support networks	Brotherhood	*“Talk about it with other men because that’s what we don’t do.”* NBBM, 65 yr old, CaP survivor (TH1)
	Community	*“But if there is anything I have learned, it is that this is not something you go through alone. And you have to be aware of people around you and people who are invested in you, and you have to find ways to let them in as much as you can. It means as much to them to be there as it does to you to have them there.”* NBBM, 68 yr old, CaP survivor (TH2) *“I actually helped lead a prostate cancer support group when I worked at the [redacted]. It was primarily for black veterans. And a lot of what they shared was the importance of finding other black men that they connected with or had other men in general that they shared something with*” WF, pain psychologist (TH4)
	Family	*“And so, I will say to all of you, make it a point as black men, fathers, sons, uncles, grandfathers, and great-grandfathers to have these conversations with the young men in your life. It is extremely important that you take the opportunity to discuss these things. Don’t shy away from it. Be honest. Talk about the things that you know. If not, sit down and help them research together. That will help us in the black community be better for each other and keep this thing called family strong. And, again, take the opportunity to work on something that is obviously affecting us from a community perspective to make it better.”* CBBM, 64 yr old, CaP survivor (TH2) *“…first test yourselves or encourage your spouse, your brothers, and all males in your lives to do the PSA and take the necessary examinations your urologist or physician would recommend to you, and most importantly, become a part of this important brotherhood.”* NBBM, 71 yr old, CaP survivor (TH1) *“…my wife is on me with checkups and making sure. So, I don’t know. It’s important to have her there with me as someone to lean on for support.”* NBBM, 66 yr old, CaP survivor (TH4)
Culturally competent care		*“One thing that we do like to talk about is cultural competency. You know, there’s only about probably 5%…again, it depends on the state. I was in [redacted] before this. It’s even lower… for physicians who are black. Right?”* NBBM, radiation oncologist (TH3) *“So, maybe there’s not a black urologist near you, but a black urologist two hours away can advocate for someone for you, represent you, and still have a phone conversation. And that’s the other thing. It’s often the decision-making that’s important. Right? The surgical and radiation skills are important, but it’s the decision-making that you want. So, getting a virtual visit is a reasonable thing that doesn’t require resources, and often people will engage with you on that.”* NBBM, radiation oncologist (TH2) *“…knowledge is power. Understanding this, knowing what it means, what the results mean, and the opportunity for you to do various things, from radiation to surgery, is very important. So I think, you know, as far as anybody’s life is concerned, having the knowledge and understanding of what this means to an African American individual is really, really important.”* NBBM, 66 yr old, CaP survivor (TH4) *"For black men diagnosed with cancer, navigating a world lacking cultural competence is challenging. Often, there's pressure to rush into treatments or follow a predetermined path.”* ABBM, 69 yr old, CaP survivor (TH3) *“…when we look at just African American men or black men, we see as you start to tack on more social needs or the downstream effects of social determinants of health, is you add more and more, mortality gets worse. As you add more barriers to care, barriers to quality care, outcomes are worse.”* NBBM, urologic oncologist (TH4)
Decision-making	Waiting	*“You know? Part of the challenge we have is that we are all silent about it until we either hear of the grave illness of the individual or we get a death announcement. I think it’s time we start acting earlier*.*”* ABBM, 69 yr. old, CaP survivor (TH1) *“I know as a Caribbean man, one of the things that’s a drawback with Caribbean men, it’s a cultural thing, prostate rectal examination because they’re very homophobic.”* CBBM, 71 yr old, CaP survivor (TH2)
	Acting	*“Part of taking action and doing something about the issue of prostate cancer includes advocating for yourself and others through legislation in order to make access easier.”* NBBM, 65 yr old, CaP survivor (TH3)
	Mental health	*“Access is also important when discussing the mental health of patients with prostate cancer because the resources available to patients largely depend on what insurance will cover.”* BF, spouse of CaP survivor (TH4) *“Mentally, physically, you know, emotionally. And so, but I saw a difference in him. You know, just that he had cancer. All that he wanted to do was get it out. Get it out, get it out, get it out. So that’s how it impacted us originally. The fear, anxiety, cancer. Coping with it, what has helped me was to get an understanding of what happens after the fact.”* BF, spouse of CaP survivor (TH3) *“Facing cancer can be terrifying, and receiving reassurance and support from loved ones is essential for mental well-being. Reassurance can take various forms, including ‘support,’ ‘words of encouragement,’ and ‘counseling alongside medical care.’ Dealing with the mental aspect of prostate cancer can be challenging due to societal pressures. However, ultimately, we have nothing to prove to anyone but ourselves, as ‘this is not something that you go through by yourself’.”* ABBM, 69 yr old, CaP survivor (TH3) *“This also is an opportunity to openly communicate their feelings and know that others are supportive, encouraging, and concerned about their overall wellbeing. Receiving mental health services is not only good for the cancer patient, but it’s also beneficial to the caretaker and other family members. … Stigma and misinformation on mental illness can also discourage treatment. Myths that men, if they show their emotions, are weak are all factors in why they don’t receive treatment. We can’t force treatment. In order for them to receive treatment, they must be willing to be vulnerable enough to admit that they need help.”* BF, spouse of CaP survivor, psychologist (TH2)
Decisional regret		*“*…*I spoke with his urologist. The doctor who was going to do the surgery was asking him about the intimacy part of it. That we never had a problem. He wasn’t having any problems before he was diagnosed, and I didn’t even understand why he took the test. You’re not having a problem; why are you taking the test? But I understand now, after listening to Dr. [redacted]. So I asked the doctor about it, and his comment to me was that if he’s not having a problem… sexual problem before surgery, he won’t have any after. So, I’m okay. … I believed the doctor. But it was totally, you know, the opposite of that.”* BF, spouse of CaP survivor (TH3) *“I wish that we had investigated a little bit further and understood some of the things that were going to happen after the fact.”* NBBM, 72 yr old, CaP survivor (TH4) *“The one problem I have with the descriptions of what happens after surgery is they call erectile dysfunction a side effect. I say it’s not a side effect. I said it is an effect. It happens. And I wish that they would drop the term side and just be straightforward and say, this is going to be the effect if you have a radical prostatectomy.”* NBBM 65 yr old, CaP survivor (TH1)

*CaP* prostate cancer, *NBBM* native-born Black male, *CBBM* Caribbean-born Black male, *ABBM* African-born Black male, *BF* Black female, *WF* White female, *TH1* town hall 1, *TH2* town hall 2, *TH3* town hall 3, *TH4* town hall 4.

#### Information

Men, their families, and healthcare providers considered access to and utilization of information about CaP as forms of self-care and community-level advocacy. Participants felt informed and confident when they accessed specialist care and obtained a second opinion. They felt empowered when given high-quality information to support care decisions. Participants identified time and resources, like insurance coverage and accessible primary care, as facilitators of information gathering. Inadequate information was described as a precursor to decisional regret.

As participants collected information, they wanted to share it with their support networks. Information had individual and community-wide utility. Participants also emphasized the importance of a second opinion as valuable for decision-making.

Town halls were considered informational resources; participants sought information about alternative approaches like diet and supplements, discussed CaP symptoms, shared information about their personal experiences, and advocated for CaP screening.

#### Support Networks

Participants and panelists encouraged men with CaP to draw on external support, including brotherhood (i.e., men with CaP), family, community, and healthcare providers. An essential outcome of accessing support was reassurance, especially given the gendered cultural expectations experienced by men. Despite difficulties asking for support, participants and their families felt that *“this [CaP] is not something that you go through by yourself”* (TH2, spouse of survivor).

***Brotherhood****.* Participants felt that CaP united them in both formal and informal brotherhood support. This brotherhood was rooted in shared experiences that fostered commonality and a built-in support system and sometimes developed into supportive friendships.

***Family****.* Families provided emotional support and motivated participants to seek screening and treatment. While participants sometimes struggled to request support due to cultural hesitancy around discussing health concerns, they did encourage one other to discuss their experiences with their families. One spouse described overcoming discomfort with discussing physical intimacy and preserving the connection with her husband, who was undergoing CaP treatment. Participants expressed a collective resolve to break the generational silence surrounding family health histories and a duty to discuss their diagnosis with the family and provide mentorship related to the CaP experience.

***Community****.* Survivors often felt called to share their experiences with other BM. They described overcoming stigma and initial discomfort in discussing CaP and beginning to participate in education and advocacy within their communities.

***Healthcare providers***. Participants and panelists discussed the importance of finding a relatable healthcare provider. The ability to have frank and open discussions was considered to improve information gathering and decision-making and to reduce the impacts of racism and bias within the healthcare system.

#### Culturally Competent Care

Panelists highlighted the importance of culturally competent care. An oncologist stated, *“…someone that… shares a common background… can change the experience [of] treatment (TH3, oncologist).”* Panelists described CaP disparities between BM and NHWM, identifying racism, socioeconomic factors, and resulting chronic stress as causes, highlighting the *“huge role that ethnicity plays in just how they [men with CaP] traverse the healthcare system” (TH3, MO).* Participants’ desire for a healthcare provider who treats them like family echoed the importance of a culturally competent provider. Participants and panelists discussed the impact of racism on information—both its quality (e.g., inadequate representation of BM in medical research) and its accessibility (e.g., barriers to BM experience accessing care).

#### Decision-Making

Patient decision-making, which was influenced by the availability of information, support networks, and culturally competent care, had four primary outcomes: waiting, acting, considering mental health, and experiencing decisional regret.

***Waiting.*** Participants who waited to seek routine CaP screening or care were influenced by two factors: (1) barriers to access, including lack of insurance/providers, fear of medical checkups, and discomfort with the digital rectal examination (DRE) related to cultural beliefs stigmatizing same-sex attraction; and (2) lack of knowledge (e.g., being unaware that CaP may be asymptomatic). The discussion emphasized how waiting can compound a lack of information and lead to urgency, and that encouragement from loved ones helps prevent waiting.

***Action.*** Action covered testing, screening, and treatment, including active surveillance. Participants recounted the various tests and treatments accessed from screening through survivorship, and both participants and panelists emphasized using the multiple available screening strategies to ease discomfort surrounding the DRE. Due to barriers like scarcity of urologists and high treatment costs, participants considered advocacy, including legislative advocacy, to be action.

***Mental health*.** Participants were aware of the stigma of living with a mental health condition and of seeking mental health support. Survivors spoke to the pressure associated with embodying a masculine role and the need to provide financially for their families and conceal “weakness.” Both panelists and participants discussed the interdependencies between mental and physical health and encouraged the utilization of mental health services.

***Decisional regret*.** Participants and spouses experienced decisional regret when they lacked information or time to decide on treatment, often feeling pressure to act immediately. Fear and anxiety from a cancer diagnosis amplified the pressure to choose between treatment plans they knew little about. An oncologist urged participants to take their time, seek a second opinion/specialist information, and read external treatment guidelines to combat decisional regret.

## Discussion

Between 2022 and 2023, four virtual town halls were conducted focusing on CaP diagnosis and management, psychosocial support, and overall survivorship to create a collaborative environment to address CaP-related challenges and solutions. Attendees included Black individuals from American, African, and Caribbean backgrounds, reflecting the diversity and complexity within the Black community [16] and underscoring the importance of inclusive outreach efforts in addressing CaP disparities.

Feedback revealed that the town halls were well received and lauded for their importance, organization, and quality. Over 40% of survey respondents were CaP survivors, signifying the relevance of these town halls to those directly impacted by CaP and the importance of providing support and resources tailored to survivors’ needs. The attendance of caregivers, family, and community members also suggests the potential for similar events to facilitate sharing experiences and foster solidarity within this community.

BM are disproportionately affected by CaP [17], experiencing more aggressive disease and higher mortality. These disparities have multifaceted determinants, including biological, socioeconomic, and healthcare related [17, 18]. Town halls echoed these concerns, emphasizing the importance of tailoring survivorship strategies to the specific needs of ethnically diverse BM [3, 4]. Transitioning from treatment to survivorship was underscored as a critical period significantly impacting QoL, necessitating tailored survivorship care strategies [7, 18, 19]. By acknowledging diversity within the Black community, interventions and support mechanisms can better address the unique challenges faced by Black CaP survivors.

The town halls highlighted three major themes—information, support networks, and culturally competent care—that critically influenced patient care decisions' timing, content, and emotionality. Access to comprehensive information was crucial; participants and oncologists emphasized seeking second opinions, reviewing current treatment recommendations, discussing treatment options and potential impacts with support networks, and not rushing treatment decisions. This aligns with previous research highlighting the significance of knowledge sharing in bolstering community health outcomes [11, 20, 21].

The role of support networks, including family, community, and healthcare providers, resonates with other findings suggesting BM prefer interpersonal communication, including from trusted sources like physicians and other cancer survivors, for CaP information [22]. This underscores the criticality of both information and the channel through which it is conveyed. Support networks provide a foundation of trust and understanding, fostering an environment where men feel more comfortable discussing and taking action related to CaP treatment.

Culturally competent care emerged as a central theme, reflecting how ethnicity and cultural beliefs shape health attitudes and behaviors [23, 24]. Participants stressed the importance of healthcare providers who understand and respect their cultural backgrounds, emphasizing the need for inclusive and culturally sensitive approaches to care delivery. This aligns with existing research highlighting the role of cultural competence in improving health outcomes and reducing healthcare disparities [24, 25]. Promoting cultural competency among healthcare providers and institutions can enhance patient–provider communication, trust, and treatment adherence.

Decision-making is influenced by the interplay of information, support networks, and culturally competent care, leading to diverse outcomes, including waiting, acting, and decisional regret. Participants discussed barriers to action, like access to care and mental health stigma, that could be reduced through a comprehensive approach to CaP management, including advocacy for better healthcare policies and resources [15]. Acknowledging decisional regret among survivors and their families points to the critical need to ensure timely, informed, and supported decision-making in CaP care. The inclusion of healthcare professionals, researchers, and community advocates provided a comprehensive support network, bridging gaps between patients and the medical community and highlighting the critical role of patient education in navigating the complex CaP journey [8].

The study had some limitations. The sample primarily consisted of individuals with access to and comfort in using online meeting platforms, potentially limiting the representation for those with limited internet access or technological literacy. Additionally, reliance on self-reported survey data may introduce response bias, impacting the generalizability of the findings. Moreover, town halls might not have fully captured all segments within the Black community, particularly those from rural or lower socioeconomic backgrounds, potentially overlooking specific nuances in experiences and needs.

Despite these limitations, the town halls shed light on critical themes: the importance of comprehensive information, robust support networks, and culturally competent care in CaP management. Future research should strive for broader inclusivity by considering diverse socio-cultural contexts to develop tailored interventions effectively addressing CaP disparities.

## Conclusion

Town halls are a powerful platform for engaging BM in discussions about CaP survivorship, shedding light on their unique experiences, challenges, and needs. The collective wisdom shared during these events highlights the importance of community engagement, culturally competent care, and strong patient–provider relationships in addressing health disparities faced by BM. Future research should build upon these findings to develop targeted interventions that promote informed decision-making, improve access to care, and enhance the overall QoL of BM with CaP. Town halls allowed Black CaP survivors to provide peer support and education while accessing culturally relevant information from healthcare providers. Given their effectiveness in community engagement and education [11], town halls should remain integral to broader CaP advocacy and support programs. Additionally, they can be adapted for addressing other health conditions affecting underrepresented populations, supporting a holistic healthcare approach that encompasses physical, psychological, and social well-being.

## Supplementary Information

Below is the link to the electronic supplementary material.

Supplementary file1 (DOCX 546 KB)

## Data Availability

Data for this project is available upon request from the corresponding author.
