# Supplementary material for: Bridging the Gap: Enhancing Prostate Cancer Survivorship and Advocacy Among Ethnically Diverse Black Men Through Community Town Halls
Source: J Cancer Educ. 2025 Feb 24;40(6):865–74. doi: 10.1007/s13187-025-02587-1 (PMC12717206; doi:10.1007/s13187-025-02587-1)
Supplement: Supplementary file 1 — Supplementary file1 (DOCX 546 KB) [file 13187_2025_2587_MOESM1_ESM.docx]

**ONLINE RESOURCE**

**Table 1: Virtual Town Hall Meeting Agenda**

**Date and Time:** Thursday, November 30^th^, 6 pm CDT/7 pm EDT

**Topic:** Prostate Cancer Survivorship in Black Men: Elevating Comfortability Around Health Conversations and Early Diagnosis

Agenda

| Time | Agenda Item | Purpose/Detail |
| --- | --- | --- |
| 6.00 pm | Consent Form | - Emphasize the voluntary nature of the meeting and the use of recordings and chats for data analysis/validation. - Inform participants about the risks and benefits of participation and that the event will be recorded. |
| 6.00 - 6:05 pm | Welcome Speech | - Reminder about survey link and incentives for participation. - Introduction of the moderator. |
| 6:05 - 6:20 pm | Keynote Speaker (Urologic Oncologist) | - Overview of prostate cancer, early detection, and treatment options.   Addressing cancer risk susceptibility and comfortability surrounding health conversations. |
| 6:20 - 6:50 pm | Panel Discussion (Black Survivors’ Perspectives) | - Personal stories, decision-making, and the importance of regular screenings. - Overcoming stigma, building support networks, and inspiring resilience. - Creating comfortable health conversations within families. |
| 6:50 - 7:00 pm | Mental Health Advocate Perspective (Psychologist) | - Importance of mental health wellness on physical wellbeing. - Highlighting mental health resources available to Black men. |
| 7.00 - 7:25 pm | Q&A Session | - Interactive session where participants asked questions and engaged with speakers. |
| 7:25 - 7:30 pm | Closing Remarks | - Summary and highlights of talks - Thank you and conclusion |

**Figure 1: Virtual Town Hall Meeting Flyer**


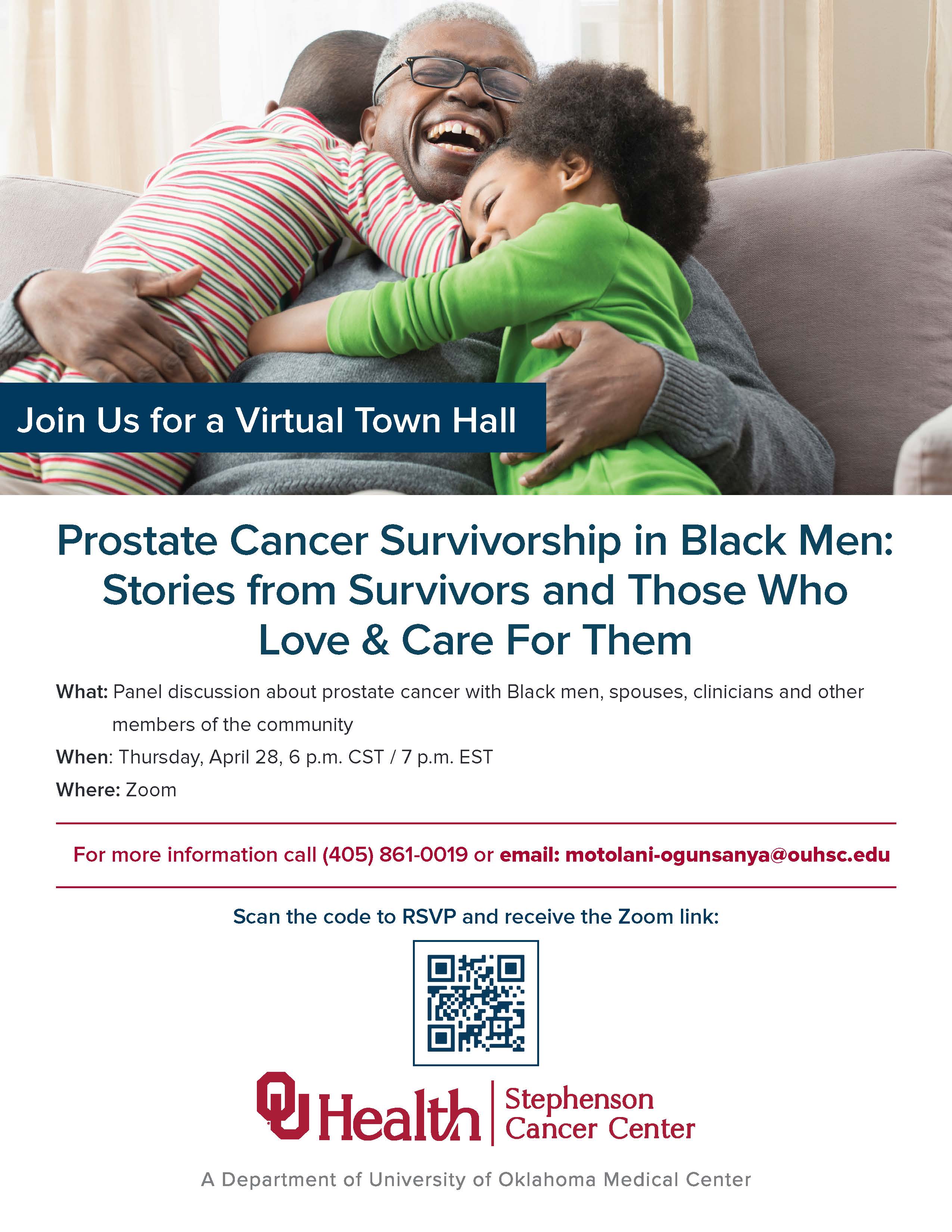


**Table 2: Summary of Survey Topics and Subtopic**

| Survey Topic | Subtopics/Questions |
| --- | --- |
| Demographic Information | Marital status, annual household income, ethnic background, country of origin and duration lived in the us |
| Feedback on the Townhall Event | Attendance, overall rating, organization, likes/areas of improvement/missing information, helpfulness of speakers' presentations, perceived community benefit, reason for attending, suggested changes, future topic preferences, how they learned about the event. |
| Prostate Cancer Diagnosis and Treatment* | Family history, relationship to prostate cancer, age at diagnosis, cancer stage, current treatment status and types |
| Prostate Cancer Experience* | Cultural factors (Blackness, masculinity, taboos), coping strategies, fear of recurrence, substance use, treatment decision-making, access to care and health literacy, relationships with providers, support circles, advocacy, and risk communication, post-treatment complications and quality of life, spirituality and religiosity |
| Follow-Up | Willingness for further contact, name, gender, email address (optional) |

*For prostate cancer survivors only
